# Exploring the flavour structure of the high-scale MSSM

**DOI:** 10.1140/epjc/s10052-020-7821-1

**Published:** 2020-03-31

**Authors:** Gino Isidori, Sokratis Trifinopoulos

**Affiliations:** Physik-Institut, Universität Zürich, 8057 Zurich, Switzerland

## Abstract

We analyse the sensitivity of quark flavour-changing observables to the MSSM, in a regime of heavy superpartners. We analyse four distinct and motivated frameworks characterising the structure of the soft-breaking terms by means of approximate flavour symmetries. We show that a set of six low-energy observables with realistic chances of improvement in the near future, namely $\Delta M_{s,d}$, $\epsilon _K$, $\epsilon _K'/\epsilon _K$, $\mathcal {B} (K\rightarrow \pi \nu {{\bar{\nu }}})$, and the phase of *D*–${\bar{D}}$ mixing, could play a very important role in characterising these frameworks for superpartner masses up to $\mathcal {O}(100)$ TeV. We show that these observables remain very interesting even in a long-term perspective, i.e. even taking into account the direct mass reach of the most ambitious future high-energy colliders.

## Introduction

The Minimal Supersymmetric extension of the Standard Model (MSSM) is one of the most motivated and attractive ultraviolet completions of the Standard Model (SM). The absence of direct signals of new particles at the LHC have pushed the scale of Supersymmetry (SUSY) breaking in the few TeV regime (or above), making the MSSM a less natural solution to the electroweak hierarchy problem. However, many other virtues of the MSSM, such as gauge coupling unification, a natural dark-matter candidate, and the possibility of coherently embedding gravitational interactions in the context of supergravity, remains open if the SUSY breaking scale is up to $\mathcal {O}(100)$ TeV. The MSSM with soft-breaking terms in this energy domain provides also a successful prediction for the Higgs boson mass [1–5], as well as natural a-posteriori justification for the heaviness of the top mass, responsible for the radiative breaking of the electroweak symmetry [6–10].

An additional important virtue of high-scale MSSM is a minor tension with flavour-physics observables, or a less severe SUSY flavour problem (see e.g. [11–14]). However, a residual flavour problem remains and flavour-physics observables may represent the only option to test the MSSM in the near future, if the SUSY breaking scale lies is in the 10–100 TeV domain. Such high-scale breaking, which is quite motivated given the argument listed above, would indeed prevent direct signals of super-partners at the LHC, even during the forthcoming high-luminosity phase, while indirect signals may still be within the reach of existing facilities. The purpose of this paper is to provide a quantitative estimate, and a detailed comparison, of the sensitivity of selected flavour-physics observables up to $\mathcal {O}(100)$ TeV.

The fact that MSSM could, in general terms, induce sizeable deviations from the SM in specific flavour-changing observables, such as $\epsilon _K$, even with squark masses above 100 TeV, is quite clear from the general sensitivity studies in the literature (see e.g. [11, 12]). What is less obvious are the following three questions: (1) does this statement remains true under realistic hypothesis about the flavour structure of the soft-breaking terms, able to justify (at least to some extent) why we have not seen any deviation form the SM yet? (2) Which low-energy observables –with realistic chance of improvements in the near future– are still interesting, given the existing tight constraints on most of them? (3) Do flavour observables remains interesting even in a long-term perspective, i.e. taking into account direct mass reach on squarks of future colliders such as the FCC-ee and the FCC-hh [15, 16]. These are the basic questions we aim to address in the present study.

To achieve this goal, we consider four basic hypotheses about the flavour structure of the soft-breaking terms: the Minimal Flavour Violation (MFV) hypothesis [17–19], a chiral *U*(2) flavour symmetry [20–22], a *U*(1) symmetry a la Froggatt–Nielsen [23, 24], and finally the framework of disoriented *A* terms [25]. As we shall discuss, implementing these different hypotheses leads to a MSSM with a different degree of tuning in the flavour sector. However, it is fair to state that all of them address in a satisfactory way the residual SUSY flavour problem, i.e. provide a reasonable a-posteriori justification of why we have not seen yet any non-standard effect at low energies.

Within these four basic frameworks we analyse a set of representative $\Delta F=2$ and ${\Delta F=1}$ flavour-changing observables in the quark sector. Restricting the attention to those where we can expect significant improvements on the experimental and/or the theoretical side, we address the pragmatic question, if a significant ($3\sigma$) deviation from the SM –compatible with present constraints– can be generated within the MSSM. We address this question as a function of the soft-breaking scale, determining this way the maximal scale at which each of the flavoured versions of the MSSM listed above can be tested indirectly via low-energy flavour-changing observables.

## Flavour structure the soft-breaking terms

### Generalities

We consider the MSSM with *R* parity conservation, whose soft-breaking terms can be divided into three categories,

(i) mass terms for the scalar fields:
  1 $$\begin{aligned}&- m_{H_U}^2 H_i^{U*} H_i^U - m_{H_D}^2 H_i^{D*} H_i^D - (m_{\tilde{Q}}^2)_{IJ} \tilde{Q}_i^{I*}\tilde{Q}_i^{J}\nonumber \\&\quad - (m_{\tilde{U}}^2)_{IJ} \tilde{u}_R^{J *}\tilde{u}_R^{I} - (m_{\tilde{D}}^2)_{IJ} \tilde{d}_R^{J *}\tilde{d}_R^{I}\nonumber \\&\quad -(m_{\tilde{L}}^2)_{IJ} \tilde{L}_i^{I*}\tilde{L}_i^{J} - (m_{\tilde{E}}^2)_{IJ} \tilde{e}_R^{+ I * }\tilde{e}_R^{+ J}. \end{aligned}$$
(ii) mass terms for the gauginos:
  2 $$\begin{aligned} \frac{1}{2} M_1 \lambda _B\lambda _B + \frac{1}{2} M_2 \lambda _A^i\lambda _A^i + \frac{1}{2} M_3 \lambda _G^\alpha \lambda _G^\alpha + \text {h.c.} \end{aligned}$$
(iii) trilinear couplings of the scalar fields:
  3 $$\begin{aligned}&\epsilon _{ij} (A_U)_{IJ} H_i^U \tilde{Q}_{j}^I \tilde{u}_R^{J *} + \epsilon _{ij} (A_D)_{IJ} H_i^D \tilde{Q}_{j}^I \tilde{d}_R^{J *} \nonumber \\&\quad + \epsilon _{ij} (A_L)_{IJ} H_i^D \tilde{L}_{j}^I \tilde{e}_R^{J *} + \text {h.c.} \end{aligned}$$

Beside the two Higgs mass terms and the three gaugino masses, the vast majority of soft breaking terms (hence of the free parameters of the model) are encoded in the $3 \times 3$ complex matrices $m_{\tilde{Q}}^2$, $m_{\tilde{U}}^2$, $m_{\tilde{D}}^2$, $m_{\tilde{L}}^2$, $m_{\tilde{E}}^2$, $A_U$ and $A_D$, characterising the flavour structure of the model.

Since we assume the overall scale of the soft-breaking terms to be significantly higher that the electroweak scale, it is a good approximation to neglect the electroweak corrections to the physical masses of squarks and leptons. This allow us to establish a simple connection between soft-breaking terms (i.e. Lagrangian parameters) and the $6\times 6$ squark and slepton mass matrices. For example, in the case of the up squarks, the $6\times 6$ mass matrix reads

4 $$\begin{aligned} \tilde{M}_{U}^2 \approx \left( {\begin{array}{*{20}{c}} (\tilde{M}_{U}^2)_{LL}&{}(\tilde{M}_{U}^2)_{LR} \\ (\tilde{M}_{U}^2)_{LR}&{}(\tilde{M}_{U}^2)_{RR} \end{array}} \right) , \end{aligned}$$

where

5 $$\begin{aligned} (\tilde{M}_{U}^2)_{LL}&=m_{\tilde{Q}}^2, \nonumber \\ (\tilde{M}_{U}^2)_{LR}&= v_U A_U, \nonumber \\ (\tilde{M}_{U}^2)_{RR}&=m_{\tilde{U}}^2. \end{aligned}$$

Similar relations holds for the down-type ($\tilde{M}_{D}^2$) and the slepton ($\tilde{M}_L^2$) mass matrices.^1^

A completely generic (flavour-anarchic) structure for the soft-breaking terms leads to strong tensions with various flavour-changing neutral-current (FCNC) and of CP-violating observables, even for sfermion masses well above 10 TeV (see e.g. [11–14]). This is why in the following we consider four different hypothesis about the underlying flavour structure of the theory able to provide a consistent (a posteriori) justification of why the flavour off-diagonal entries in (4), and the other sfermion mass matrices, are suppressed.

### Minimal flavour violation

The starting point of the MFV hypothesis is the flavour group $\mathcal {G}_{F}$,

6 $$\begin{aligned} \mathcal {G}_{F}&= \mathcal {G}_{q} \times \mathcal {G}_{l} \nonumber \\ \mathcal {G}_{q}&= U(3)_{Q} \times U(3)_{U} \times U(3)_{D}, \ \ \ \ \ \ \mathcal {G}_{l} = U(3)_{L} \times U(3)_{E}, \end{aligned}$$

corresponding to the flavour symmetry that the SM Lagrangian enjoys in the limit of vanishing Yukawa couplings. The basic assumption is that the only quantities that break this symmetry are spurion fields proportional to the SM Yukawa couplings themselves. In the MSSM context this implies that the soft-breaking terms can be reconstructed entirely out of appropriate powers of the SM Yukawa matrices $Y_{U,D,E}$ [19].^2^ Keeping only the leading terms in the expansion in terms of flavour-changing spurions leads to a significant reduction in the number of free parameters [26, 27]. Furthermore, without affecting the phenomenological analysis (focused on quark flavour-changing observables), we assume real gaugino masses and universal slepton masses. According to these hypotheses, we end up with a total of 14 parameters characterising all the soft-breaking in this framework:

7 $$\begin{aligned}&M_1, \ \ M_2, \ \ M_3 \nonumber \\&(\tilde{M}_{Q}^2)_{IJ} = \tilde{m}_Q^2 \left( \delta _{IJ} + x_1 V_{3I}^*V_{3J} \right) , \nonumber \\&(\tilde{M}_{U}^2)_{IJ} = \tilde{m}_U^2 \left( \delta _{IJ} + x_2 \delta _{3I} \delta _{3J} \right) , \ \ \ (\tilde{M}_{D}^2)_{IJ} = \tilde{m}_D^2 \delta _{IJ} \nonumber \\&(\tilde{M}_{L}^2)_{IJ} = \tilde{m}_L^2 \delta _{IJ}, \ \ \ (\tilde{M}_{E}^2)_{IJ} = \tilde{m}_E^2 \delta _{IJ} \nonumber \\&(A_U)_{IJ} = a_0 \delta _{3I}V_{3J}, \nonumber \\&(A_D)_{IJ} = a_0 \left( \delta _{3I} \delta _{3J} + y_5 \delta _{3I} V_{3J} \right) , \ \ \ (A_E)_{IJ} = a_0 \delta _{IJ}. \end{aligned}$$

The $x_i$ and the overall soft masses $\tilde{m}_{Q/U/D}^2$ and $\tilde{m}_{L/E}^2$ are necessarily real, while the remaining parameters can be complex. By consistency of the framework, the dimensionless parameters $x_i$ and $y_i$ are restricted to be at most of $\mathcal {O}(1)$ [19].

### *U*(2) chiral flavour symmetry

An approach quite similar to the MFV hypotheses, but based on a smaller flavour symmetry and a larger set of symmetry-breaking terms, is obtained assuming a flavour symmetry acting only on the light generations. The basic premise of this hypothesis is the observation that *U*(2) flavour symmetries provide a good description of the SM Yukawa couplings, explaining the smallness of light-fermion masses compared to third-generation ones. Restricting the attention to the quark sector, we employ the following flavour symmetry [22]

8 $$\begin{aligned} \mathcal {G}_{q} = U(2)_Q \times U(2)_U \times U(2)_D, \end{aligned}$$

acting on the first two generations. We further assume that the symmetry is broken by three spurions: the leading spurion $V_Q$, responsible for breaking the left-handed flavour subgroup $U(2)_Q$ (or the left-handed mixing between third- and light-generations),^3^ and the sub-leading spurions $\Delta Y_{U(D)}$, transforming as bi-doublets of $U(2)_{U(D)} \times U(2)_Q$. In terms of these symmetry breaking terms, the quark Yukawa couplings can be written as9where $y_{t,b}$ are the third-generation Yukawa couplings, and $x_{t,b}$ are $\mathcal {O}(1)$ parameters.

When describing flavour-mixing in the soft-breaking terms, we neglect the subleading $\Delta Y_{U(D)}$ spurions, which are very small as a consequence of the smallness of light-quark masses. In this limit flavour mixing appears only in the left-handed sector. Employing the explicit parametrization given in [22], one may express the squark mass matrix in terms of a small number of mixing angles and masses:

10 $$\begin{aligned} \tilde{M}_{Q}^2&= W_L^d \times \text {diag} (\tilde{m}_{Q_h}^2, \tilde{m}_{Q_h}^2, \tilde{m}_{Q_l}^2) \times W_L^{d \dagger }, \nonumber \\ \tilde{M}_{U}^2&= \text {diag} (\tilde{m}_{u_h}^2, \tilde{m}_{u_h}^2, \tilde{m}_{u_l}^2), \ \ \ \tilde{M}_{D}^2 = \text {diag} (\tilde{m}_{d_h}^2, \tilde{m}_{d_h}^2, \tilde{m}_{d_l}^2), \nonumber \\ A_U&= a_0, \ \ \ A_D = a_0 y_b. \end{aligned}$$

where

11 $$\begin{aligned} W_L^d = \left( {\begin{array}{*{20}{c}} c_d&{}\kappa ^*&{}-\kappa ^* s_L e^{i\gamma }\\ -\kappa &{}c_d&{}-c_d s_L e^{i\gamma }\\ 0&{}s_L e^{-i\gamma }&{}1 \end{array}} \right) . \end{aligned}$$

The parametric size of the parameters appearing in the left-handed mixing matrix $W_L^d$ is as following:

12 $$\begin{aligned} c_d \sim \mathcal {O}(1), \quad \kappa =c_d \frac{V_{td}}{V_{ts}} \sim \lambda \equiv |V_{ts}|, \quad \left| s_L e^{\pm i\gamma }\right| \sim \lambda ^2.\nonumber \\ \end{aligned}$$

To further simplify this framework, and also to distinguish it from the general MFV case discussed above, we further assume the limit where the first two generations of squarks are very heavy. This hypothesis is not an exclusive requirement of the *U*(2) flavour symmetry, but it is a particularly interesting case considered in the literature (the so-called effective SUSY or split-family SUSY [28, 29]), which cannot be achieved under the MFV hypothesis and which is particularly motivated given the present lack of direct SUSY signals at the LHC (see e.g. [30] and references therein). Under this additional hypothesis our *U*(2) framework is fully characterised by the entries in $W_L^d$, the universal trilinear term $a_0$, and by the light third-generation masses ($\tilde{m}_{Q_l}^2$, $\tilde{m}_{u_l}^2$, $\tilde{m}_{d_l}^2$, and corresponding terms for the sleptons).

### *U*(1) Froggatt–Nielsen

As representative example of a framework with larger flavour-violating terms, we consider the holomorphic *U*(1) Froggatt–Nielsen symmetry acting on the quark sector proposed in [24]. In order to successfully reproduce the observed SM mass hierarchies, the quarks (and correspondingly to the squarks) are assigned the following $U(1)_\mathrm{FN}$ charges:

13 $$\begin{aligned}&Q_{L 1,2,3} \sim (3,2,0), \ \ \ u_{R 1,2,3}^c \sim (3,2,0), \nonumber \\&d_{R 1,2,3}^c \sim (1,0,0). \end{aligned}$$

The symmetry is spontaneously broken via one single familon $\theta$ carrying a positive $U(1)_\mathrm{FN}$ charge $Q_\theta =+1$. For instance, the up-Yukawa coupling takes the form

14 $$\begin{aligned} \epsilon _{ij} Y_U^{IJ} H_i^U Q_{j}^I (u^c)^J = \epsilon _{ij} \left[ a_{IJ} \left( \frac{\theta }{M}\right) ^{u_J+q_I}\right] H_i^U Q_{j}^I (u^c)^J,\nonumber \\ \end{aligned}$$

where $a_{IJ}$ are coefficients of $\mathcal {O}(1)$, $\theta$ denotes the familon VEV, $q_I$, $u_I$ are the charges defined in (13) and *M* the cut-off scale of the effective theory. Setting $\epsilon = \theta / M \approx \lambda$ one obtains a good description of the SM Yukawa coupling (with suitable choices of the $\mathcal {O}(1)$ parameters).

Proceeding in a similar manner for the soft-breaking terms in the squark sector we arrive to the following parametric decomposition (we omit to write the unknown $\mathcal {O}(1)$ complex coefficients):

15 $$\begin{aligned} \tilde{M}_{Q}^2&= \tilde{m}_Q^2 \left( {\begin{array}{*{20}{c}} 1&{}\epsilon &{}\epsilon ^3\\ \epsilon &{}1&{}\epsilon ^2\\ \epsilon ^3&{}\epsilon ^2&{}1 \end{array}} \right) , \ \ \ \tilde{M}_{U}^2 = \tilde{m}_U^2 \left( {\begin{array}{*{20}{c}} 1&{}\epsilon &{}\epsilon ^3\\ \epsilon &{}1&{}\epsilon ^2\\ \epsilon ^3&{}\epsilon ^2&{}1 \end{array}} \right) ,\nonumber \\ \tilde{M}_{D}^2&= \tilde{m}_D^2 \left( {\begin{array}{*{20}{c}} 1&{}\epsilon &{}\epsilon \\ \epsilon &{}1&{}1\\ \epsilon &{}1&{}1 \end{array}} \right) , \nonumber \\ A_U&= a_0 \left( {\begin{array}{*{20}{c}} \epsilon ^6&{}\epsilon ^5&{}\epsilon ^3\\ \epsilon ^5&{}\epsilon ^4&{}\epsilon ^2\\ \epsilon ^3&{}\epsilon ^2&{}1 \end{array}} \right) , \ \ \ A_D = a_0 \left( {\begin{array}{*{20}{c}} \epsilon ^4&{}\epsilon ^3&{}\epsilon ^3\\ \epsilon ^3&{}\epsilon ^2&{}\epsilon ^2\\ \epsilon &{}1&{}1 \end{array}} \right) . \end{aligned}$$

The $\mathcal {O}(1)$ complex coefficients of the $\mathcal {O}(\epsilon ^n)$ terms in these matrices, together with their overall normalisation, represent the free parameters of this framework. As in the MFV case we assume universal slepton masses.

### Disoriented *A* terms

The disoriented *A*-term framework [25] is a scenario exclusive to SUSY where flavour violation occurs only in the *L*–*R* mixing terms, hence the SM Yukawa and the trilinear soft-breaking terms. However the latter are not assumed to be aligned, as opposed to the MFV case. More precisely, the soft-breaking mass terms in (1) are assumed to be exactly diagonal, whereas the *A* terms can be decomposed as

16 $$\begin{aligned}&(A_{F})_{IJ} = A_0 \theta _{IJ}^{F} y_{F_J}, \ \ \ \ F=U,D \nonumber \\&(A_{E})_{IJ} = A_0 \theta _{IJ}^{E} y_{E_J}. \end{aligned}$$

where $\theta _{IJ}^{F/E}$ are generic $\mathcal {O}(1)$ mixing angles. The latter, together with the universal soft masses, are the free parameters of this framework.

The possibility of the absence of flavour violation in the soft masses (quadratic terms), together with a sizeable flavour mixing in the *A* terms is not unlikely in supersymmetric theories: a scenario of this type can be realised, for instance, if a non-abelian flavour symmetry act on the *R*-invariant part of the supersymmetry-breaking terms, ensuring (total or partial) universality of soft masses, but is violated in the *R*-charged sector, allowing for general trilinear terms. It is also worth to stress that the separation between soft masses and trilinear interactions of the first two generations is quite robust under the renormalization-group flow, making this scenario technically stable. Last but not least, the experimental bounds on the $\theta _{IJ}^{F/E}$ are indeed of $\mathcal {O}(1)$, even for squark masses of a few TeV [25] making this scenario quite consistent from the phenomenological point of view.

## Flavour-changing observables

In this Section we enumerate the flavour-changing observables taken into account in our phenomenological analysis, illustrating their key features both in the SM and in the MSSM. On general grounds, the number of potentially interesting flavour-changing observables is quite large. However, we restrict the attention to a relatively small number according to the following main criterium: we consider observables which provide, at present, a stringent constraint on the MSSM (in flavour space) and/or can be expected to be significantly improved in the near future. Moreover, we focus only on lepton-flavour conserving observables.

According to this criterium, we restrict the attention to all the accessible $\Delta F=2$ observables and to three selected $\Delta F=1$ transitions, namely $\epsilon _K'/\epsilon _K$, $\mathcal {B} (B\rightarrow X_s\gamma )$ and $\mathcal {B} (K^+ \rightarrow \pi ^+ {\bar{\nu }} \nu )$. The latter are representative of the three most-relevant classes of relevant flavour-changing amplitudes: non-leptonic ones ($\epsilon _K'/\epsilon _K$), FCNC with leptons ($K^+ \rightarrow \pi ^+ {\bar{\nu }} \nu$) and FCNC with photons ($B\rightarrow X_s\gamma$).^4^

The current values of these observables are shown in Table 1. In the last column of this table we show a hypothetical future scenario where, thanks to (realistic) improvements on the experimental and/or the theoretical side, some these observables could exhibit a significant deviation from the SM. These hypothetical future results will be used in our numerical analysis to assess the sensitivity of each observable to the high-scale MSSM.

A more detailed discussion of the various observables follows below.

**Table 1:** Experimental values and SM predictions for the observables used in the numerical analysis (see main text)

Observable	Experiment [36]	$O_\mathrm{exp}/O_\mathrm{SM} - 1$	Future scenario $(3\sigma )$
$\Delta M_{B_d}$	$(0.5064 \pm 0.0019)~\mathrm{ps}^{-1}$	$-0.13 \pm 0.09$ [37, 38]	$-0.13 \pm 0.04$
$\Delta M_{B_s}$	$(17.757 \pm 0.021)~\mathrm{ps}^{-1}$	$-0.12 \pm 0.07$ [37, 38]	$-0.12 \pm 0.04$
$|\epsilon _K|$	$(2.229\pm 0.010) \times 10^{-3}$	$0.10 \pm 0.09$ [39]	$0.10 \pm 0.03$
$\mathcal {B}(B\rightarrow X_s\gamma )$	$(3.52\pm 0.25)\times 10^{-4}$	$0.11 \pm 0.11$ [40]	–
		$$O_\mathrm{exp}- O_\mathrm{SM}$$	
$\epsilon _K'/\epsilon _K$	$(16.6 \pm 2.3) \times 10^{-4}$	$(11 \pm 7)\times 10^{-4}$	$(11 \pm 3.6)\times 10^{-4}$
$\mathfrak {I}(M^D_{12})/M_D^2$	$(0.0 \pm 4.6)\times 10^{-17}$ [39]	$(0.0 \pm 4.6)\times 10^{-17}$	$(4.6 \pm 1.5)\times 10^{-17}$
$\mathcal {B}(K^+\rightarrow \pi ^+\nu {{\bar{\nu }}})$	$(0.85 \pm 0.5)\times 10^{-10}$ [41, 42]	$(0.0 \pm 0.5)\times 10^{-10}$	$(0.3 \pm 0.1)\times 10^{-10}$
$\Delta M_K/M_K$	$7.0 \times 10^{-16}$	$(0 \pm 7) \times 10^{-16}$	–

- $\Delta F = 2.$ If quark- and lepton-flavour violating interactions occur independently and without a special chiral structure, $\Delta F = 2$ amplitudes naturally provide the most stringent constraints on heavy scale new-physics (see e.g. [43, 44]). In the $B_d$ and $B_s$ systems both magnitudes and phases of the meson-antimeson mixing amplitudes are dominated by short-distance dynamics and are experimentally determined with high accuracy. In order to minimise the impact of the current SM uncertainties, we implement the corresponding constraints normalising the experimental results to SM predictions. On the phases, we can expect only marginal improvements in the data/theory comparison before being saturated by irreducible theory errors. On the other hand, significant room for improvement can be expected on the magnitudes, thanks to improved Lattice-QCD predictions for $\Delta M^\mathrm{SM}_{d(s)}$ (see e.g. [37, 38]).
  The situation is opposite in the Kaon and *D*-meson systems, where only the phases (or better the CP-violating mixing amplitudes) are short-distance dominated. The potential reduction of the error in $\epsilon _K$ reported in Table 1 is quite optimistic but not unrealistic in the long-term. As pointed out in [45], right now the perturbative error in $\epsilon _K$ is $\sim 3\%$, a similar error is due to non-perturbative parameters (whose precision is expected to improve thanks to improved Lattice-QCD analyses), and the dominant error ($\sim 7\%$) is due to parametric uncertainties (associated mainly to $|V_{cb}|$ and $|V_{ub}|$). The latter are expected to decrease significantly in the next few years thanks to measurements at both LHCb and Belle-II.
  Despite the lack of a precise SM prediction for $\Delta M_K$, we implement a constraint based on $\Delta M_K$ requiring non-standard contributions to this observable not to exceed, in magnitude, the SM short-distance contribution estimated in [46]. As far as CP violation in *D*-meson mixing is concerned, we implement the constrain on $\mathfrak {I}(M^D_{12})$ from [39] without normalising it to the SM expectation (assuming the latter to be negligible).
  The MSSM contributions to the $\Delta F = 2$ observables are induced by squarks–gauginos box diagrams. The dominant contribution usually comes from the gluino-squark box, whose expression in the case of degenerate squarks can be found in [11]. Depending on the spectrum of the gaugino masses, chargino-squark boxes can also become sizable. The latter can be found in [47].
- $\epsilon _K'/\epsilon _K.$ The strong suppression of $\epsilon _K'/\epsilon _K$ within the SM, which is not only due to the CKM hierarchy but also to an accidental SM low-energy property (the so-called $\Delta I=1/2$ rule), makes this observable a particularly sensitive probe of non-standard sources of both CP and flavour symmetry breaking. The main difficulty here is the uncertainty on the SM prediction. The value we report in the table is an educated guess taking into account the recent results in [48–51]. For a conservative implementation of the present constraint on new physics, we adopt an inflated error covering all available predictions within the $1\sigma$ interval. In the future scenario we illustrate how the situation could change if new lattice-QCD data on the hadronic matrix element, which so far are still affected by large errors [52], would clearly establish a deviation from the SM.
  Within the MSSM there are four distinct amplitudes able to generate sizable contributions to $\epsilon _K'/\epsilon _K$: isospin-breaking gluino-squark box diagrams [53] (also known as Trojan penguins [54]), chargino-squark box diagrams [55] and chargino-mediated [56, 57] as well as gluino-mediated [58] *Z*-penguin diagrams (relevant to scenarios with sizeable trilinear terms).
- $\mathcal {B} (B\rightarrow X_s\gamma )$. Historically $\mathcal {B} (B\rightarrow X_s\gamma )$ has been one of the most significant constraint on the structure MSSM [59], as well as many other new physics models. We base our constraint on the precise SM result in [40] which has an error comparable to the experimental one. Given irreducible uncertainties associated to non-perturbative effects, this error will hardly improve in the next few years.
  The MSSM constraint is obtained expanding the SM NNLO expression for the $B\rightarrow X_s\gamma$ rate in terms of the Wilson coefficients of the dipole ($C_7$) and chromo-magnetic dipole ($C_8$) operators around their SM central values, as in [60]. SUSY corrections are then obtained computing $\Delta C_{7(8)} = C^\mathrm{MSSM}_{7(8)} -C^\mathrm{SM}_{7(8)}$ using the one-loop chargino photon- and gluon-penguin amplitudes in [59].
- $\mathcal {B} (K^+ \rightarrow \pi ^+ {\bar{\nu }} \nu )$. This rare process may provide one of the most clean and stringent constraint on flavour-changing *Z*-penguin amplitudes. Contrary to $\mathcal {B} (B\rightarrow X_s\gamma )$, here the dominant error is the experimental one, which is expected to significantly improve in the near future. We base our present constraint on the old BNL-E949 experimental result [41], and the very recent NA62 result presented at KAON 2019 [42]. Averaging the two we obtain the value reported in Table 1, whose central value coincides with the most up-to-date SM prediction [61].
  The relevant MSSM contributions include chargino and gluino *Z*-penguin diagrams with non-negligible, $SU(2)_L$-breaking trilinear couplings as well as chargino boxes. The former yielding the dominant contribution at high energies [62].

## Numerical analysis and discussion

### Analysis strategy

As illustrated in Table 1, the observables are divided into two categories depending if they are normalised to the SM or not. In these two groups we define the expected deviations from the SM as

17 $$\begin{aligned} \Delta O_i^\mathrm{NP}(x) = \left\{ \begin{array}{l} O_i^\mathrm{NP}/O_i^\mathrm{SM} - 1 \\ O_i^\mathrm{NP}- O_i^\mathrm{SM} \end{array} \right. \end{aligned}$$

where $O_i^\mathrm{NP}$ denotes the observables in the MSSM, and *x* generically denotes the corresponding parameters (soft masses) in a given MSSM implementation. To compute explicitly the $\Delta O_i^\mathrm{NP}(x)$ we use the expressions of the observables in terms Wilson Coefficients of dimension-six operators according to the following references:

- $\Delta B = 2$ and $\Delta S=2$ amplitudes: Eqs. (7.24)–(7.27) in Ref. [63];
- $\Delta C = 2$ amplitude: Eq. (3) in Ref. [64];
- $\epsilon _K'/\epsilon _K$: Eq. (82) in Ref. [65],
- $\mathcal {B} (B\rightarrow X_s\gamma )$: Eq. (33) in Ref. [60];
- $\mathcal {B} (K^+ \rightarrow \pi ^+ {\bar{\nu }} \nu )$: Eq. (24) in Ref. [62].^5^

The expression of the Wilson Coefficients in the MSSM are computed at the one-loop level, taking into account all the amplitude discussed in Sect. 3. The corresponding loop functions are expanded around the diagonal entries of the squark mass matrices (mass insertion approximation) up to second non-trivial order for each observable (see Appendix in [56]). The entries in the diagonal are kept non-degenerate. We have explicitly cross-checked our analytic one-loop expressions with the corresponding degenerate limits reported in the literature (see references listed in Sect. 3).

In the third column of Table 1 we report the present constraints on the $\Delta O_i^\mathrm{NP}(x)$, taking into account current experimental results and SM estimates. We assume a Gaussian distribution with mean value $\mu _i$ and standard deviation $\sigma _i$ (including both experimental and theoretical errors). In the fourth column we report hypothetical $\{\mu ^F_i, \sigma ^F_i\}$ corresponding to a possible $3 \sigma$ deviation from the SM compatible with present data. Since no significant improvement is expected in the near future on the SM predictions for $\Delta M_K$ and $\mathcal {B} (B\rightarrow X_s\gamma )$, no future scenario is considered for these two observables.

**Fig. 1:**
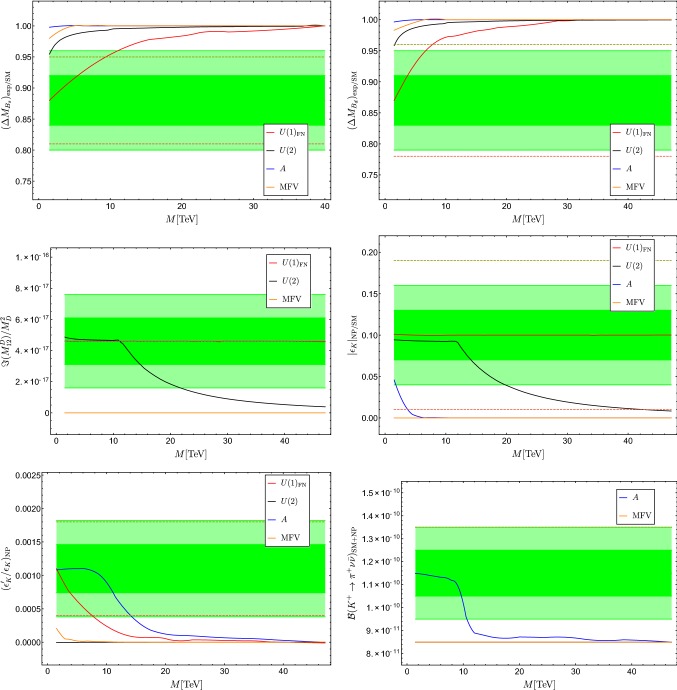
Comparison of different flavour models, within the MSSM, in accommodating observables that could exhibit a $3 \sigma$ deviations with respect to the SM, as function of the overall soft-breaking scale *M* (see main text). The green bands denote the $1 \sigma$ and $2 \sigma$ regions around the central values in the hypothetical future scenario for each observable, while the dashed lines denote the present $1 \sigma$ range. When a given flavour models does not appear on a given plot, it is implied that it yields a completely negligible contribution to the corresponding observable

Our goal is to scrutinize the capability of each flavour model of Sect. 2 to provide a best-fit point that improves over the SM setting one of the observables to the future scenario, while keeping all the others to the current values. For each scenario we thus construct six different $\chi ^2$ functions,

18 $$\begin{aligned} \chi _i^2(x)= & {} \left( \frac{\Delta O_i^\mathrm{NP}(x) - \mu ^F_i}{\sigma ^F_i}\right) ^2+ \sum _{j\not =i} \left( \frac{\Delta O_j^\mathrm{NP}(x) - \mu _j}{\sigma _j}\right) ^2;\nonumber \\&\qquad \qquad i=1,\ldots ,6, \end{aligned}$$

depending on which observable is set to the value in the fourth column. The $\chi _i^2(x)$ are then minimized as a function of the model parameters. In each case we minimize the $\chi ^2$ function setting all SUSY masses around an overall scale *M*, requiring the trilinear couplings to be compatible with the vacuum stability bounds [66]. As a convention, we choose *M* to be the mass of the third generation squark and we allow the other masses to vary within one order of magnitude around this scale (except for the heavy squark masses in the *U*(2) case, which are always assumed to be decoupled), checking the compatibility with bounds from direct searches. We start from the current lower bounds for the masses in [36] and repeat the procedure for increasing values of *M* until we eventually reach the point of decoupling. For each observable we interpolate between the consecutive best-fit points of each flavour model and different values of *M* obtaining the continuum lines shown in Fig. 1.

As far as the dimensionless parameters of the models are concerned, they are varied within their natural range as indicated in Sect. 2. More precisely, the absolute size of the $\mathcal {O}(1)$ parameters is allowed to vary between 1/10 and 4 in the MFV and *U*(2) cases, and between 1/10 and 1 in the other two. A special care is required for the $U(1)_\mathrm{FN}$ model, in the regime $M <10$ TeV: here the $\mathcal {O}(1)$ parameters controlling first-second generation mass insertions need to be tuned at the per-cent level (or even per-mil level, for the lowest values of *M*) in order to satisfy the relevant low-energy bounds. In order to present results for this model at low *M* we allow this tuning. Moreover, to clearly distinguish the $U(1)_\mathrm{FN}$ model from the disoriented *A*-terms scenario, we set off-diagonal trilinear couplings to zero in the former case.

### Discussion

The lines interpolating the consecutive best-fit points for the six flavour observables as a function of *M*, in the different flavour models, are shown in Fig. 1. The shapes of the lines suffer from some minor numerical instabilities. Still, they provide a good illustration of the main findings of this analysis, which can be summarised as follows:

- **Decoupling.** With the only exceptions of $|\epsilon _K|$ and $\mathfrak {I}(M^D_{12})$, i.e. of the $\Delta F=2$ mixing in the 1–2 sector, for all other observables and for all flavour models, the decoupling limit is reached for *M* well below $50~\mathrm{TeV}$. On the other hand, in each observable there is at least one flavour model able to accommodate a significant deviation from the SM for *M* below 10 TeV.
- **MFV vs.** ***U(2)***. In these two scenarios flavour mixing in the MSSM is closely connected to the structure of the CKM matrix. Interestingly enough, the two frameworks behave quite differently: while the MFV framework is unable to accommodate sizeable effects in any observable, within the *U*(2) model large deviations from the SM can occur in $\mathfrak {I}(M^D_{12})$ and $|\epsilon _K|$. This difference can be understood by the rigidity of the MFV framework, where large off-diagonal entries in the squark mass matrices are necessarily accompanied by an overall increase of squark masses. Moreover, in the *U*(2) model under consideration there is an effective decoupling of the mixing between third generation and first two generations vs. the mixing among the first two generations [22]. This feature allows sizeable effects in $\mathfrak {I}(M^D_{12})$ and $|\epsilon _K|$ that are not restricted by constraints from other observables.
  Nevertheless, both in MFV and in the *U*(2) model the absence of large non-diagonal *A* terms and the constraints from $\Delta B=2$ amplitudes prevent large effects in the $\Delta F=1$ observables.
- ${{\varvec{U(1)}}}_{\mathbf{FN}}$. As expected, the $U(1)_\mathrm{FN}$ model is the one which can accommodate large deviations from the SM, even at relatively large *M* values, in most of the observables. The only observable where this does not occur is $\mathcal {B} (K^+ \rightarrow \pi ^+ {\bar{\nu }} \nu )$, but this is because of our particular implementation of the $U(1)_\mathrm{FN}$ model. As a matter of fact, with non-universal *A* terms as expected in general (e.g. see Eq. 15), this framework can accommodate large deviations in $\mathcal {B} (K^+ \rightarrow \pi ^+ {\bar{\nu }} \nu )$ similarly to those shown in the disoriented *A*-term case.
  It is worth stressing that in the $|\epsilon _K|$ and $\mathfrak {I}(M^D_{12})$ cases the $U(1)_\mathrm{FN}$ model provides a good fit even for very large values of *M*: the decoupling for $\mathfrak {I}(M^D_{12})$ occurs at $M\sim 300~\mathrm TeV$ and at even higher energies for $|\epsilon _K|$. As anticipated, the price to pay for this ‘flexibility’ is the tuning of the model parameter at low energies.
- **Disoriented** ***A*** **terms**. This flavour model is the one accommodating the largest effects in the two $\Delta F=1$ observables, at fixed *M*. This is because of the potentially large impact in down-type $\Delta F=1$ amplitudes induced by chargino-mediated *Z*-penguin diagrams. Interestingly, and somehow unexpectedly, the disoriented *A*-term framework provides a (slightly) better fit to $\epsilon _K'/\epsilon _K$ with respect to the $U(1)_\mathrm{FN}$ one. As in the case of $\mathcal {B} (K^+ \rightarrow \pi ^+ {\bar{\nu }} \nu )$ mentioned above, this is a consequences of our illustrative choice of neglecting *A*-term contributions in the $U(1)_\mathrm{FN}$ case. The large difference between the tiny effects in $\Delta F=2$ observables vs. the large effects in ${\Delta F=1}$ observables provides a distinctive signature of the *A* terms. This fact is a general consequence of the $SU(2)_L$-breaking nature of the *A* terms, which imply that their contribution in $\Delta F=2$ box diagrams leads to effective dimension-8 operators (with four fermion and two Higgs fields), which are strongly suppressed for $M > 1$ TeV.

Beside these specific observations, a key feature emerging from this analysis is the complementary role of these six observables in reconstructing the structure of the soft-breaking terms, in the hypothesis that one (or more) of them will exhibit a deviation from the SM in the near future. Figure 1 shows indeed that each model is associated to a characteristic signature with large or small effects, at a given scale, in a given subset of observables.

## Conclusions

The flavour structure of the soft-breaking terms has always been considered one of the most puzzling aspects of the MSSM. The necessity to raise the overall scale of SUSY breaking, in order to satisfy the null results of direct searches, has partially ameliorated the SUSY flavour problem: while with soft masses in the $\mathcal {O}(100)$ GeV regime only MFV-like scenarios were compatible with low-energy data, with a SUSY breaking scale of a few TeV (or above) many more options are allowed. However, a residual flavour problem remains in the motivated range of soft-masses below 100 TeV, which preserves many of the virtues of the MSSM. On the one hand, this motivates a non-trivial flavour structure for the soft-breaking terms. On the other hand, this implies that low-energy flavour-changing observables might represent a unique opportunity to probe the structure of the MSSM in the next few years.

In this paper we have provided a quantitative estimate of the sensitivity of selected flavour-physics observables to four distinct (and motivated) frameworks characterising the flavour structure of the soft-breaking terms for soft-masses up to $\mathcal {O}(100)$ TeV. We have shown that even in scenarios where flavour mixing is CKM-like, specifically in the case of a chiral *U*(2) flavour mixing, $\Delta F=2$ observables could exhibit deviations from the SM for superpartners as heavy as 10 TeV. In this regime the model would escape direct searches in the high-luminosity phase of the LHC, while the deviations in low-energy observables would a have realistic chances to be detected (combining improvements both on the theory and on the experimental side).

More generally, we have shown that a set of six low-energy observables with realistic chance of improvements in the near future, namely $\Delta M_{s,d}$, $\epsilon _K$, $\epsilon _K'/\epsilon _K$, $\mathcal {B} (K\rightarrow \pi \nu {{\bar{\nu }}})$, and the phase of *D*–${\bar{D}}$ mixing, could play a very important role in characterising the flavour structure of the MSSM with heavy superpartners. Our findings are summarised by the plots in Fig. 1, which illustrate the capability of each of the four flavour models to fit a realistic deviation from the SM in each of these observables, as a function of the overall SUSY breaking scale.

One of the most striking features of the six observables in Fig. 1 is their complementarity in the regime of quark masses of $\mathcal {O}(10)$ TeV, which is the regime that could be probed at the FCC-hh (see Fig. 38 in [16]). This provides a clear illustration of the importance of low-energy flavour-changing observables even in a long-term perspective. We stress that this is not a generic statement valid only in models with flavour-anarchic soft-breaking terms, which lack theoretical justification. As we have shown with our four explicit examples, it remains true also in very motivated models based on approximate flavour symmetries.

We finally note that further key low-energy constraints (and possibly hints) on the high-scale MSSM could be obtained by lepton-flavour violating observables and by the flavour-conserving electric-dipole moments of quarks and leptons [67, 68]. For the sake of simplicity, we focused the present analysis only on quark flavour-violating observables, since these are the ones for which the relevance of future high-precision measurement was less obvious. If the MSSM is the ultraviolet completion of the SM, the improvement on all these low-energy observables is a necessary tool to reconstruct its flavour structure from data.

## Data Availability

This manuscript has no associated data or the data will not be deposited. [Authors’ comment: All the data relevant to this publication are available in graphical form via the plots.]

## Footnotes

1 We assume $\tan \beta {\mathop {_\sim }\limits ^{<}}10$ and $\mu = O (v_U)$, such that $\mu \tan \beta$ terms in down-type and slepton mass matrices are negligible.

2 Without loss of generality, we work in the so-called down-basis, where the SM Yukawa couplings assume the following from: $Y_U = \lambda _U V$, $Y_D = \lambda _D$, and $Y_E = \lambda _E$. Here $\lambda _U=\text {diag}(y_u,y_c,y_t)$, $\lambda _D=\text {diag}(y_d,y_s,y_b)$, $\lambda _E=\text {diag}(y_e,y_{\mu },y_{\tau })$ and *V* is the CKM matrix.

3 The natural size of the leading spurion is $V_Q = \mathcal {O}(|V_{ts}|)$.

4 We do not include in our analysis observables related to the recent *B*-physics anomalies (see e.g. [31, 32]) since, as we have explicitly verified, there is no way to generate sizeable modifications to these observables the *R*-parity conserving high-scale MSSM. As shown in [33–35], a good description of current anomalous results in *B*-physics can be obtained in supersymmetric extensions of the SM relaxing the hypothesis of *R*-parity conservation.

5 We have contacted the authors and confirmed that the parameter $\kappa$ should be rescaled by a factor of $\left( \frac{2^{3/2} \pi \sin ^2\theta _W v}{\alpha }\right) ^2$.
